# NeuroPlace: Categorizing urban places according to mental states

**DOI:** 10.1371/journal.pone.0183890

**Published:** 2017-09-12

**Authors:** Lulwah Al-barrak, Eiman Kanjo, Eman M. G. Younis

**Affiliations:** 1 Bristol University, Computing Department, Bristol, United Kingdom; 2 Department of Computing and Technology, Nottingham Trent University, Nottingham, United Kingdom; 3 Faculty of Computers and Information, Minia University, Minia, Egypt; University of Rijeka, CROATIA

## Abstract

Urban spaces have a great impact on how people’s emotion and behaviour. There are number of factors that impact our brain responses to a space. This paper presents a novel urban place recommendation approach, that is based on modelling in-situ EEG data. The research investigations leverages on newly affordable Electroencephalogram (EEG) headsets, which has the capability to sense mental states such as meditation and attention levels. These emerging devices have been utilized in understanding how human brains are affected by the surrounding built environments and natural spaces. In this paper, mobile EEG headsets have been used to detect mental states at different types of urban places. By analysing and modelling brain activity data, we were able to classify three different places according to the mental state signature of the users, and create an association map to guide and recommend people to therapeutic places that lessen brain fatigue and increase mental rejuvenation. Our mental states classifier has achieved accuracy of (%90.8). NeuroPlace breaks new ground not only as a mobile ubiquitous brain monitoring system for urban computing, but also as a system that can advise urban planners on the impact of specific urban planning policies and structures. We present and discuss the challenges in making our initial prototype more practical, robust, and reliable as part of our on-going research. In addition, we present some enabling applications using the proposed architecture.

## 1 Introduction

The increase in stress-related illnesses is escalating dramatically in the world. Stress can be a chronic disease that is difficult to detect and is often associated with a stigma of embarrassment and humiliation. Yet, the impact of stress is profound, costing the UK economy £3.7billion per year due to work absence, and much more in inefficient task execution. Furthermore, human beings have contended with many stressors in their daily life that may cause many health problems such as increased heart rate and blood pressure and altered immune system function. Daily life activities require constant concentration and attention that might lead to fatigue and stress, as explained in the Attention Restoration Theory (ART) introduced by Kaplan [1]. Today, many people restore attention and seek relief through meditation or outdoor recreation. Nature and urban environments offer a restorative experience that may impact individuals’ well-being. However, some environments are hectic and might not relieve stress or remedy attention problems and exhaustion. Given today’s technological advances, several studies have emerged which can be utilized to assess the effects of built environments on humans using physiological sensors. For instance, heart rate monitors and skin conductivity sensors, have shown enhanced results following the exposure to restorative environments. Recently, affordable wireless Electroencephalogram (EEG) headsets capturing the electric potentials of neuronal populations have become available. Originally designed for Brain-Computer Interfaces (BCI) to assist physically impaired individuals, BCI also carries new research prospects applications in many domains. In this work, we study brain signals in an attempt to understand the effects of outdoor built environments on mental activity, and in particular: the restorative state. In addition, we provide an objective measure of how different place categories impact our mental states. This paper achieves this goal by employing low-cost EEG devices for data collection and analysis. A predictive model is then built in order to provide a better understanding of how the exposure to different outdoor environments may foster or hinder recovery from stress, the investigation also correlates the mental state with environmental acoustic noise levels. The built environments considered in this work consist of both green spaces and urban built areas, and hence allow us to know how the exposure to natural green spaces may promote greater attention restoration and stress recovery than visiting built environments. In the final part of the work, we present two classification techniques for the mental state results and visually represented on geographical maps to recommend relaxing environments for people in order to alleviate stress.

## 2 Background

### 2.1 On the pervasiveness of brain sensing

Over the past decade, many researchers have begun to explore BCI technology as a new way of communication and control for disabled people. BCI gives users the ability to communicate and control devices without depending on the normal output channels of peripheral nerves and muscles [2]. Current BCI systems use EEG activity recorded at the scalp to control devices and assist people with neuromuscular impairments. Today, many companies are offering portable and low cost EEG devices to enable the new applications of BCI [3][4][5]. The EEG devices can be connected to computers or smart phones. In this work, we used commercially available mobile EEG devices that can be used in outdoor environments to enable different emerging applications.

### 2.2 The restorative potentials of urban places

‘Place’ is defined in geographic research as “space which people have made meaningful” [6]. Perhaps more importantly, places are reproduced through people’s imaginations, memories, emotions and feelings, both positive and negative, and by using different senses [7][8]. Thrift [8] discussed place experiences during people walk in the countryside as compared to a walk in the city. The author illustrated how places are constructed through different senses and people’ s bodies. Such impressions can construct place as welcoming and pleasant or hostile and aggressive. Positive impressions about places attract people to visit these places again. For many, such places are usually quiet, restful and tranquil allowing people to reduce their stress levels and therefore remedy the Directed Attention Fatigue (DAF) by providing a palliative to the nonstop attentional demands of typical, city streets. For others, such places can be something quite different. Therefore, it is important to use data science to personalize the relationship between places and mental states. Attention restoration theory (ART) suggests that a person’s ability to direct attention in thoughts becomes fatigued with interruptions. DAF is a condition that reduces the overall mental effectiveness of the brain and results in problems in focusing and planning activities [9]. Kaplan et al. [1], Kohlleppel et al. [10] and Ulrich [11] have studied the power of natural environments in giving people restful experiences that can provide a quick and strong recovery from any stress and they found that nature provides a sense of peacefulness and tranquillity and can help in recovering from stress faster than urban environments. In addition to natural settings, coffee shops, health clubs, video arcades and some retail shops were proven to play a great role in the restoration of directed attention and can bring positive emotions and help regulating negative emotions and stress [12] [13] [14]. However, the restorative experience provided by different environments depends on many other factors such as air quality and environmental noise.

### 2.3 Environmental noise effects

**Fig 1:**
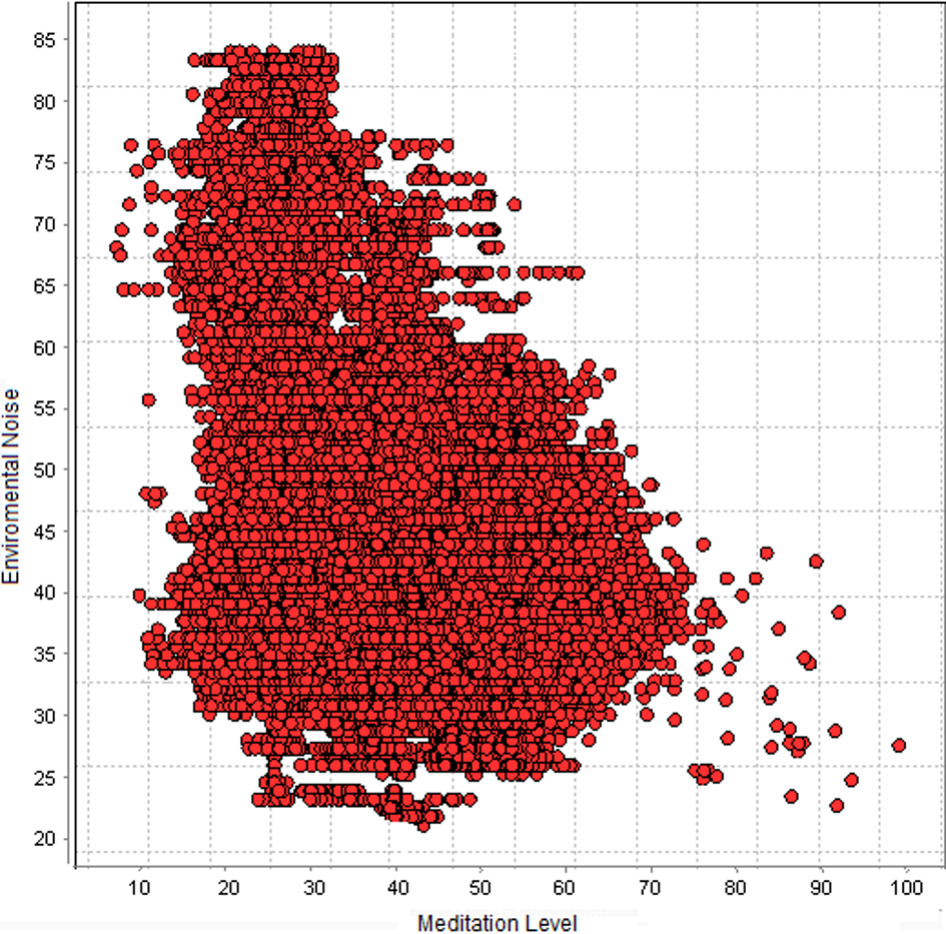
Scatter plot showing the linear relationship between meditation levels and environmental noise.

Many studies have been conducted to study the effects of environmental noise on mental health and human well-being. The research results proved that noise can impair productivity and cause serious health problems such as chronic stress and heart diseases [15]. Acoustic noise sources vary including road traffic, construction work, aircrafts, and schools, factory machinery, house-hold devices or even social celebrations. In this work, we study environmental acoustic noise due to its effects on mental states and emotions. Monitoring noise helps in detecting some abnormal environmental distractions which might affect people’ s perception of a place. Therefore, prior to studying mental states changes in outdoor places and in order to understand how high levels of environmental acoustic noise can impact our mental states, we conducted an experiment to understand the effects of high noise levels on mood and mental state. (Fig 1) shows one persons’ meditation levels collected using EEG headset in the case of both high and low acoustic noise levels for one hour. Ambient noise was recorded using Noise Spy [16], noise monitoring and mapping framework. Noise Spy allows users to explore a city area while collaboratively visualizing noise levels in real-time.

From the graph, we notice that the meditation level is decreased when acoustic noise level is high, whereas the meditation level becomes higher when the noise is low. These results indicate that environmental acoustic noise impacts our brains and can cause stress and thus change mental states. Therefore, these preliminary observations show that combining EEG data with environmental noise measurements is an indicative of correct analysis and classification of mental states associated with outdoor environments.

## 3 Related work

During the last decade, mobile sensing has drawn a lot of attention from the research community and the industry [17, 18, and 19]. Mobile sensing research includes a variety of areas, including but not limited to: health monitoring, movement tracking, carbon footprint, social pattern analysis and transportation pattern analysis. The used sensors range from physiological sensors, environmental sensors, to tracking technologies.

In a similar fashion, physiological signals contain useful patterns that help to identify individual’s mental state. For example, Healey et al. [20] have studied the changes in physiological signals such as Electrocardiogram, electromyogram, skin conductance and respiration to determine driver’s overall stress levels. They found that heart rate and skin conductivity measures were most closely correlated with stress levels. Recent studies have begun to use physiological signals to identify mood and emotions. Physiological pattern recognition of emotion has important applications in health, entertainment and human-computer interaction. Lisetti et al. [21] described their findings in relation to emotion elicitation by collecting physiological signals from subjects watching movie clips, and they found out that physiological signals can classify six different emotions with high classification accuracy.

Moreover, many applications are emerging in the area of environmental monitoring. For instance, a wearable, low power, air quality and environmental monitoring sensor has been developed by Zappi et al.[22]. The system is designed to sample air pollutant (CO, NO2 and O3). Accurate, real-time information coming from such sensors can help people who are suffering from health problems (e.g. Asthma) to avoid polluted environments[23]. Furthermore, NoiseSpy[16] is a low-cost A-weighted sound measurement system that monitors environmental noise levels, allowing users to explore a city area while collaboratively visualizing noise levels in real-time.

The relationship between emotional stability and one’s surroundings is especially important and challenging in cities, where the environment is highly varied, dynamic and densely populated. Meanwhile, the growing up-take of smart phones in the population (60% in developed countries and 15% globally) renders each device a potential data collection hub of information such as location, tweets, status updates and signal strength. Recent work has used twitter to capture the personality of an individual [24] and the general mood of a population [25][26], with applications such as stock-market prediction [27]. More recent work has shown that smartphones can be used to intervene on a user’s behaviour and habits [28][29].

The ubiquitous nature of smartphones that are coupled with cheaper sensors and increased computational power has allowed them to be considered as serious competitors to dedicated sensor platforms. For instance, Reddy et al. [30] have implemented a system that uses the phone’s GPS and accelerometer to determine the transportation mode of an individual when outside, whether the user is stationary, walking, running, biking or in motorized transport which is useful in monitoring health as well as collecting transportation data. In addition, UbiFit [31] mobile application uses accelerometer data to recognize human activities, monitoring the amount of exercise by an individual and using an unobtrusive ambient display on the phone to encourage exercising. Furthermore, Al-ajmi et al. [32] have utilized mobile phones and mobile skin conductance sensor to detect emotions in shopping malls and rate different shops which can be used to improve marketing campaigns. Also, Carriço at al. [33] have linked mobile phones to heartbeat sensors to develop a system for exposure therapy support to track patients in different environments.

EEG technology has been used mainly in BCI as a mean of communication and control to assist people with special needs. BC’s have been applied as brain machine interfaces to control wheelchair, manipulate robotic arms, or mentally write messages to allow communication in people with severe communication disorders. For instance, Yazdani et al. [34] have developed a brain-controlled wheelchair, which processes brain signals and classifies them into different control thoughts/action. However, the applications of EEG technology are not limited only to patients, healthy people can benefit from such a technology. Several studies have emerged to investigate and explore the possibilities of development in the area of Brain-Computer Interfaces using consumer friendly equipment that have recently become available on the public market. For example, Emotiv [3] and NeuroSky [4] are offering affordable and mobile EEG devices that can be used in a broad range of applications.

In [35], Wang et al. have presented a neuro-feedback game that utilizes the Emotiv EEG headset. They suggested EEG-based games can be utilized to treat mental disorders such as Attention-Deficit/Hyperactivity Disorder (ADHD) or Autistic Spectrum Disorders (ASD). BCI applications intended for people with special needs require research-grade equipment; however, NeuroPhone [36] presents an effortless, hands-free address-book dialling application, where users select a photo of a contact from the address book mentally and the application dials the chosen contact. Educational applications of EEG technology are also emerging. For instance, Mostow et al. [37] have used Neurosky EEG headsets on students to monitor cognitive processing and their mental state during learning. In addition, commercial EEG devices have opened the market to innovative entertainment applications. Mind Garden [38] is a game that utilizes the EEG technology, in whichusers train their concentration and meditation skills by playing a game. Another entertainment EEG-based brain-computer interface system developed by Wright [39], where instant message communication is made richer by attaching emotional information provided by the EEG headset since EEG headsets are capable of inferring emotions and mental states with reasonable accuracy.

These applications have been tested in indoor environments with either the EEG device is not portable or the application was developed for desktop applications. The work presented in this paper integrates mobile phones with mobile EEG headsets to offer a fully mobile experience. Debener et al. [40] have shown that good quality EEG data can be obtained in such adverse recording conditions as naturally walking outdoors. However, there is a limited amount of related work in recording EEGs data outdoors. Seigneur [41] presents a new model of economy based on the emotions that the users experience detected with mobile EEG devices. The work illustrates a tourism tour where the EEG device is set to record the events and emotions as they happen during a tour in the city. This work uses EEG devices in outdoor environments to rate places using light, mobile devices such as mobile phones and portable EEG headsets.

## 4 Materials and methods

In this section, we provide an overview of NeuroPlace system and its components. We describe each architectural component in turn, presenting a high-level view of how the system works in union to provide a scalable mobile brain sensing framework.

### 4.1 Wireless EEG headset

The recent availability of low-cost EEG headsets [3] [4] and programmable mobile phones have given researchers the ability to detect brainwaves in a ubiquitous manner. In this work, Neurosky [4] wireless EEG devices were used. Neurosky offers a variety of EEG headsets for different purposes. The prices of these devices range from $100.00 to $200.00. Neurosky products (MindWave, BrainBand) were chosen due to their affordability, portability, wireless connection capability and the availability of an open source API (Application Programming Interface). These devices transmit encrypted data over Bluetooth to mobile phones. The headsets are equipped with a single- channel EEG sensor and an electrode that rests on the forehead on the FP1 position according to the international 10–20 system and a second electrode that touches the ear [4]. They are capable of detecting raw EEG signals, frequency of different brainwaves: Delta (0–3 Hz), Theta (4–7 Hz), Alpha (8–12 Hz), Beta (12–30 Hz) and Gamma (30–100 Hz), and two mental states (attention and meditation). The attention level shows the intensity of user's level of mental "focus" or "attention". The meditation level indicates the level of "relaxation" or a user's mental "calmness". Neurosky provides mental states levels ranging from 0 to 100, where high values refer to strong engagement in the mental state and low values refer to low levels of engagement. The headset samples the raw EEG at 512 samples / sec. The frequency bands are provided at 1 Hz sampling rate, and are presented as a series of eight 3-byte long values ranging from 0 to 224. The attention and meditation mental states are also sampled at 1 sample / sec. The data rate of the EEG data streamed from the headset to the mobile phone is 250kbit per second.

**Fig 2:**
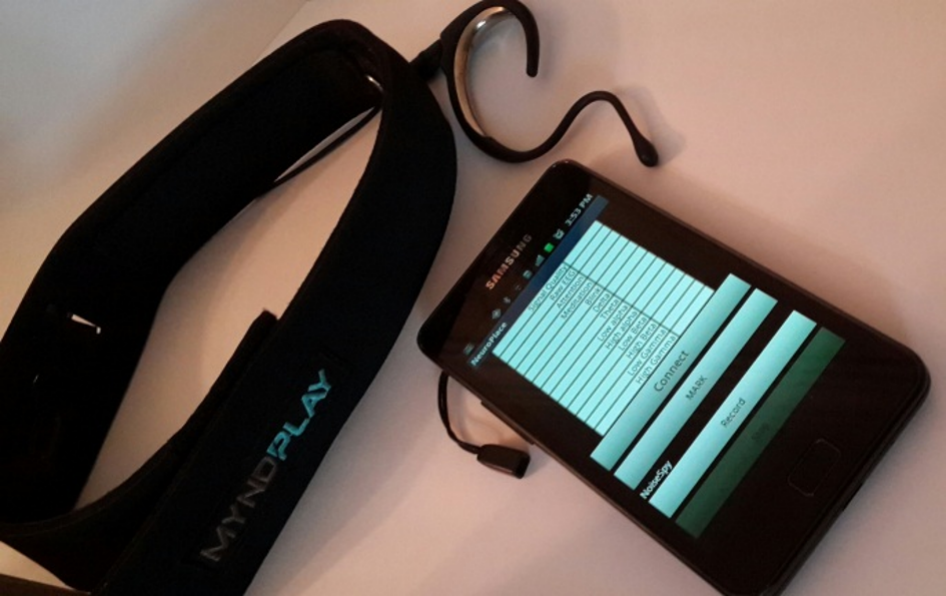
NeuroSky EEG headset and NeuroPlace mobile app.

A mobile application was developed for Android devices that connects to Neurosky EEG headsets wirelessly (see Fig 2) and record different EEG and environmental noise data in outdoor environments. The collected data are then time-stamped and saved for offline analysis.

**Fig 3:**
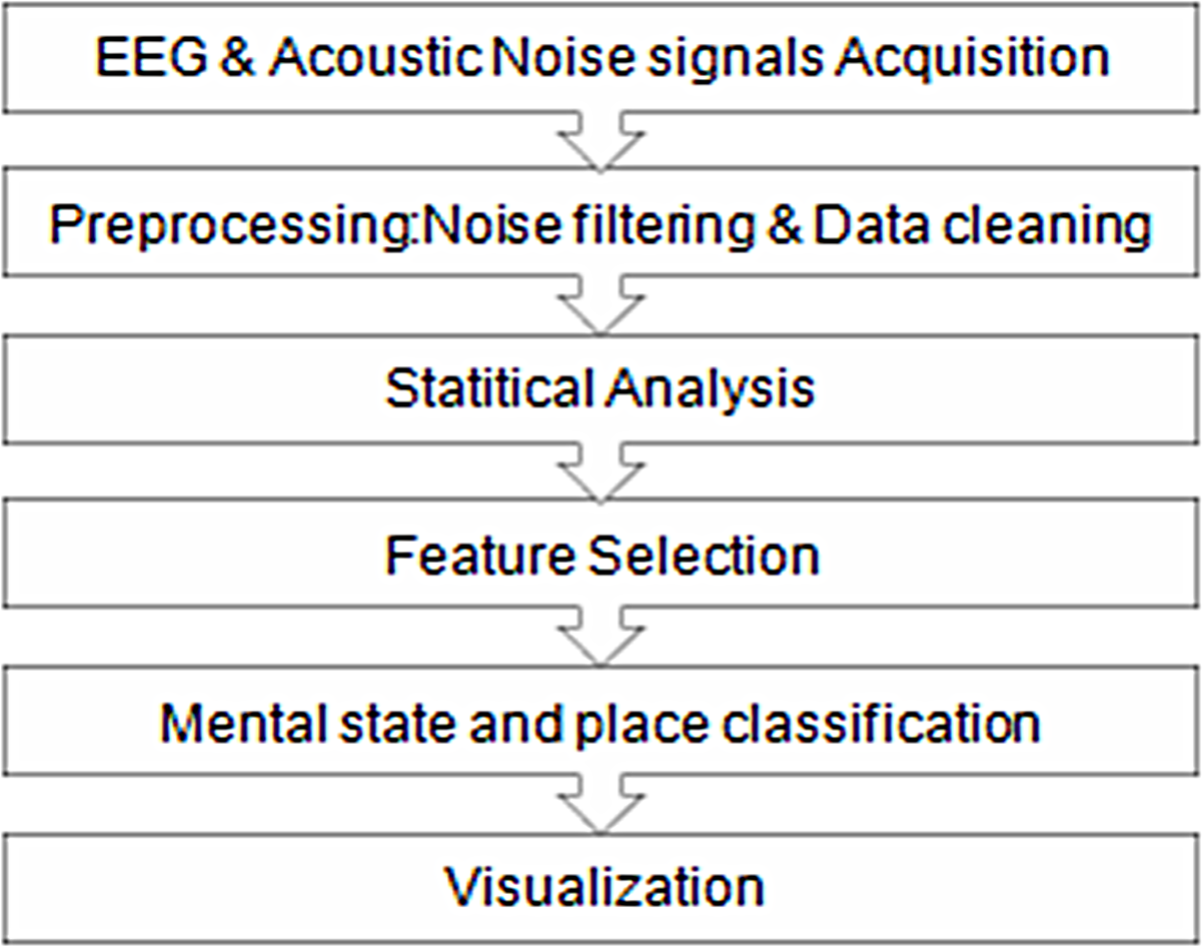
System architecture.

The work presented in this paper comprises six components as illustrated in (Fig 3). First, the EEG and environmental noise data are acquired using the EEG headset and NeuroPlace mobile application. After acquiring the data, it is necessary to pre-process the signals before analysis and classification. It is widely known that brain signals are noisy since electric potentials must pass through the skull and hence, presents challenges for signal processing and analysis. Raw EEG signals contain several artifacts such as eye blinks, cardiac signals, and muscle activity. Removal of such artifacts from EEG signals cannot be completely performed since it may result in loss of some details. Signal analysis techniques such as Independent Component Analysis (ICA) or Principal Component Analysis (PCA) have the potential to filter and isolate artifacts from the EEG signal. However, these techniques must be applied on multi-channel recordings, and hence cannot be applied on singlechannel devices such as NeuroSky headsets without modifying the aforementioned algorithms. Therefore, the work presented in this paper is based on using features calculated by NeuroSky’s algorithms to produce the eight brain frequency bands (including Delta, Theta, Low Alpha, High Alpha, Low Beta, High Beta, Low Gamma, High Gamma) and two mental states, ‘‘Attention” and ‘‘Meditation “. All of these features are referred to as‘‘EEG signals” in this paper.

These data samples are often corrupted by noise that can interfere with the quality of these signals. Baseline wandering due to head and eye movements, and muscle artifacts are major sources of distortion in EEG signal classification. In order to obtain reliable and useful information from the EEG devices, we process the EEG signal before applying any feature extraction method.

Signal filtering step is also complemented with normalizing the EEG signals according to environmental noise levels in the surrounding space.

This is due to the fact that EEG signals are affected by high level of acoustic noise in the surrounding environment (such as traffic). Pre-processing EEG signals paves the way to feature selection stage where different time and frequency domain features can be extracted and the most significant features are selected.

These steps are followed by data analysis step using One-way repeated measures ANOVA statistical technique to identify any patterns in data. The ANOVA test is often to determine whether there are any statistically significant differences between the means of three or more independent (unrelated) groups. Analysing data statistically helps in understanding and identifying the main features in the collected data for classification. Feature Selection process is followed by feature reduction in order to run and compare different machine learning techniques to classify mental states around places. Finally, the output of the place data analysis is then visualized using heat maps overlaid on a map of the selected places.

### 4.2 Experimental setup

In the following section, we discuss our experimental setup, the data analysis techniques and followed and our findings.

### 4.3 Participants and methodology

Forty participants took part in the study aged 17 to 30 (mean age of 21.5, all female). All of the participants were students. During the data collection process, we collected 672,776 lines of data. Three data files were empty and two others have some of the data fields missing. Therefore, we selected a subset of the data for analysis based on 23 users’ experiments who completed the study correctly. Therefore, our final dataset comprises of 534,346 lines of data.

The Neuroplace user study was approved by the KSU research centre and the information systems department at KSU. All users have provided a written consent to take part in the experiment.

The purpose of this study is to understand the restorative power of outdoor environments and guide people to environments are expected to calm them down. To focus on the mental behaviour of subjects in outdoor environments, we selected three distinct places that are within a walking distance from each other. Each place is perceived physiologically different environment from the other; some of them are busier and others are peaceful and tranquil. These places are:

**Caf**é: indoor and outdoor seating areas where people can enjoy coffee with a relaxing view.
**Supermarket**: a crowded mini market.
**Garden**: a small, quiet and green area with variety of trees and foliage.

Before commencing the experiment, participants were given an overview regarding the EEG technology, experiment, places and the type of data collected. Consent was signed by all of the subjects. Participants were trained on how to use the headset and the mobile application. Also a sensor warming up period was taking in account before collecting and recording the data. Participants were asked to wait few minutes before the experiment to stabilize EEG signals. Additionally, they were instructed to keep the headset still as moving the headset may cause low signal quality, and hence incorrect readings. The experiment route starts from the café, supermarket and finally the garden. In each place, the subjects were instructed to stay for five minutes and then move to the next place. The participants moved through the same sequence of places individually and were followed and observed by a researcher. All experiments were conducted during the day from 4:00 p.m. to 6:00 p.m. to avoid high weather temperatures that can cause discomfort and unpleasant feelings. One to two experiments were made per day. The total time of the experiment was approximately 20 minutes for most of the participants.

After completing the experiment, participants were asked to answer a questionnaire about their demographics, health, tag their mental state at each place explicitly and other questions to rate the device’s comfort level. The questionnaires showed that all of the subjects did not suffer from any health issues prior to the experiments. Such information is useful to understand any distinct stress patterns that may occur in the data due to these reasons.

## 5 Results

In this work, different techniques were used to analyze and detect mental states patterns in the collected data. As a start, simple plots were created to visually observe any patterns in EEG data. This was followed by the analysis of statistical significance. One-way repeated measures Analysis of Variance (ANOVA) is a statistical method used to decide whether a feature shows a significant difference between two or more classes (places) and to identify important features to classify mental states.

After that, classification of mental states was applied using two classification algorithms; Naïve Bayes and J48 Decision Trees.

### 5.1 Visual inspection

**Fig 4:**
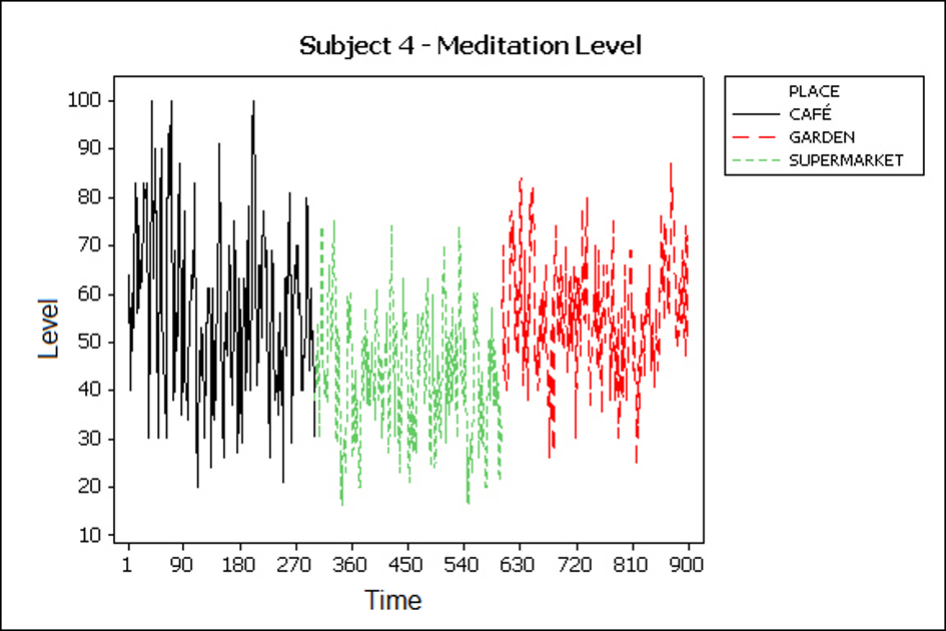
Meditation levels of participant 6.

**Fig 5:**
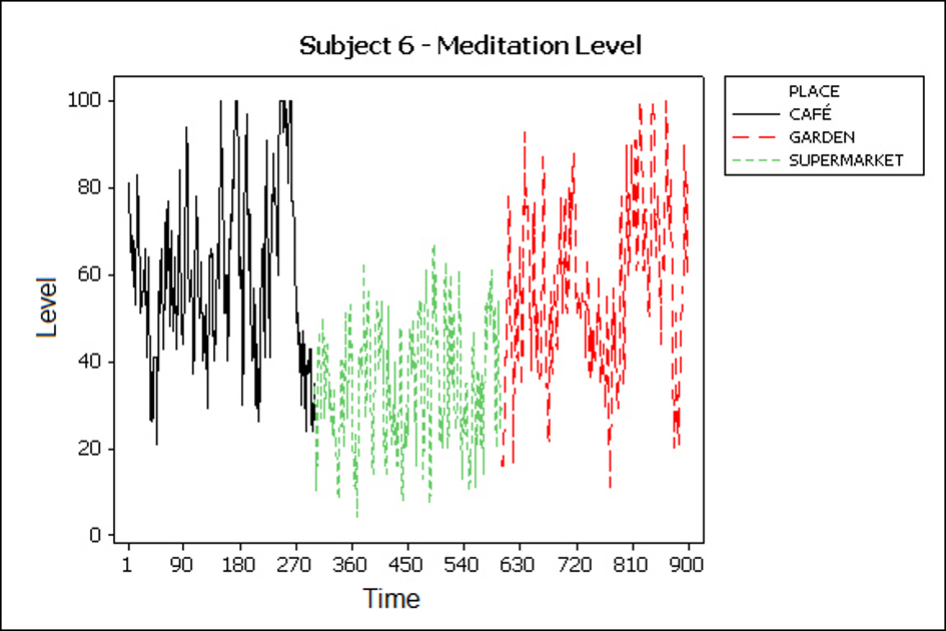
Meditation levels of participant 4.

In behaviour analysis, graphical inspection of the collected data is a standard method to evaluate it at an early stage. Meditation levels and frequency bands of subjects are plotted to illustrate the changes in levels as they move from place to place and the duration of the experiment in each place. Subjects were asked to stay at each place for 5 minutes and then move to the next place. (Fig 4) shows high meditation levels in the garden and café and lower levels in the supermarket. This happens due to the busy environment in the supermarket that may change mental state from highly relaxed to stressed. However, (Fig 5) shows a different pattern and it is noticeable that the subject was very relaxed in the café when compared with the other places.

**Fig 6:**
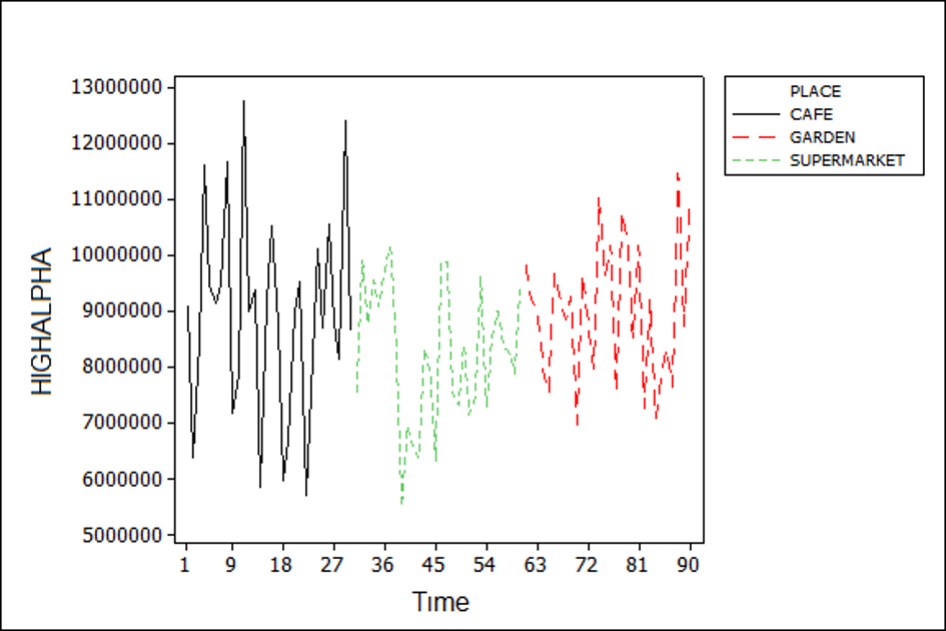
High Alpha wave of participant 6.

**Fig 7:**
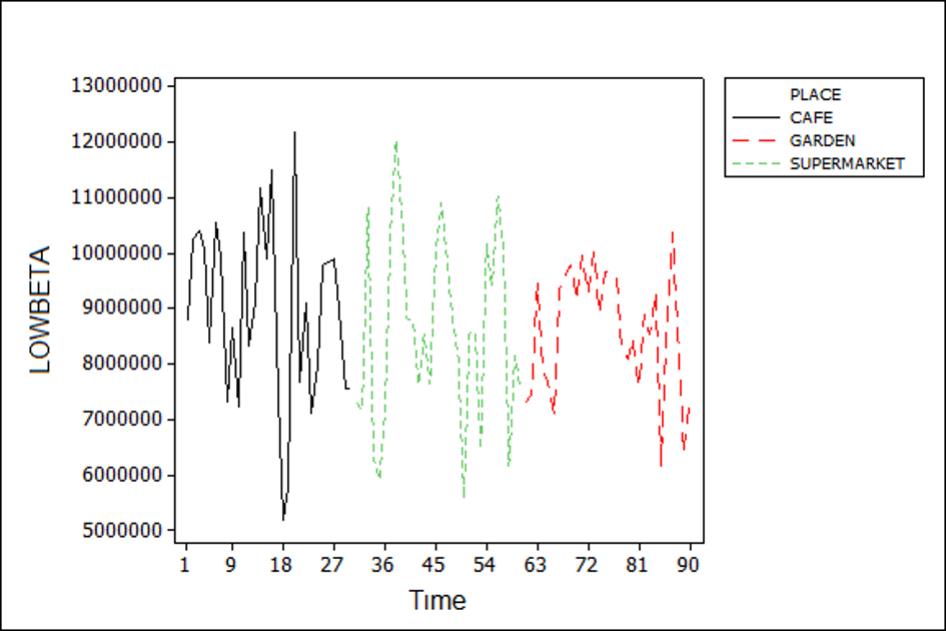
Low Beta wave of participant 6.

EEG brain waves are usually associated with specific mental state. For example, Alpha brain wave activity is generally associated with relaxed wakefulness (coherent consciousness), while Beta wave is characteristic of an engaged mind, which is highly alert and well-focused. As expected, by observing the frequency bands, it is possible to notice a distinct variation in activities among each user. (Fig 6) shows the High Alpha α band activity of the EEG data of participant 7. The graph depicts higher alpha activity in café than in the green space and garden while a low activity is noticed in the supermarket since the meditation level during the time of experiment was low. However, when we examine the Beta band β activity in (Fig 7), we observe higher activity in the supermarket and lower activity in the other places. These changes are due to the fact that the participant was focused and actively thinking in the supermarket. In returns, relaxed state in the garden is linked to lower activity of the Beta wave.

### 5.2 Statistical analysis

**Fig 8:**
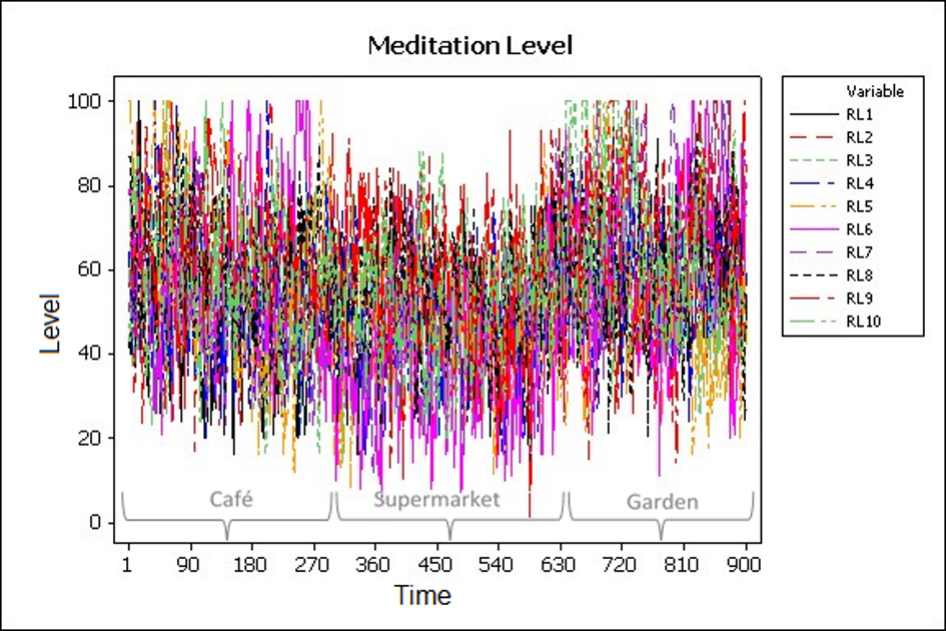
Meditation levels of 10 subjects at different places.

Statistical analysis and pattern recognition methodologies were used in this study to automatically recognize the direct impact of different urban places on the EEG signals and the associated mental states. (Fig 8) shows a clear temporal change in meditation levels in relation to the three different places (based on data extracted from the EEG headsets of ten subjects).

**Fig 9:**
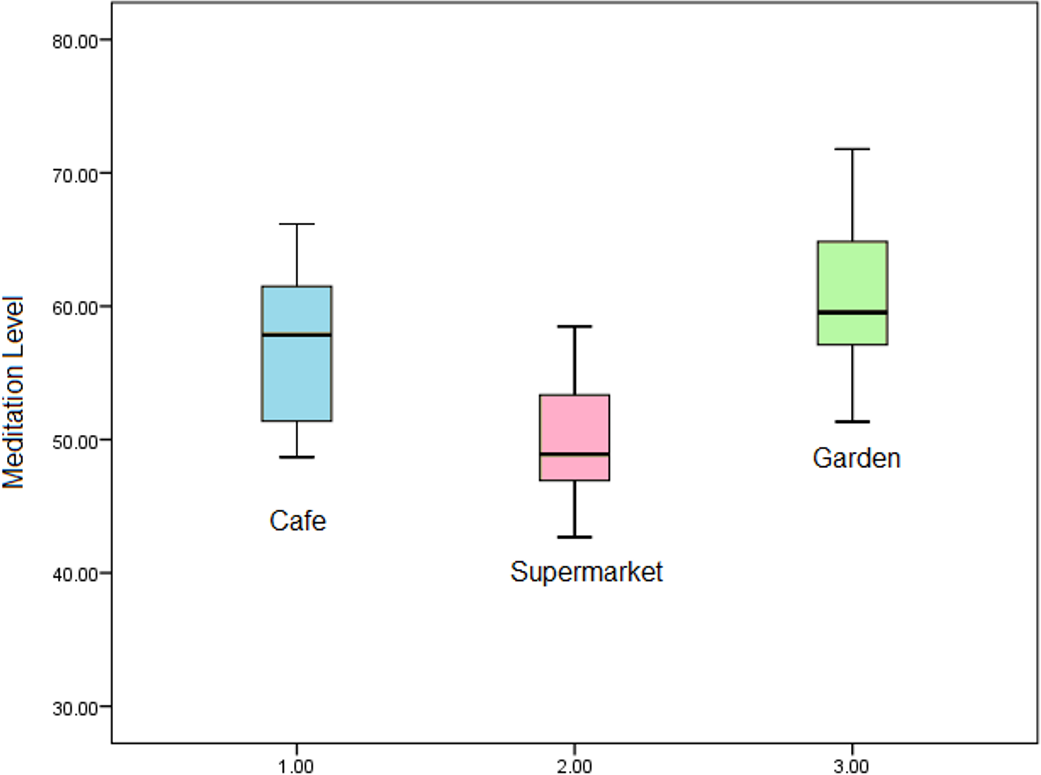
Caf*é*, supermarket and garden mediation levels.

Additionally, initial statistics describing the means *m* and standard deviation σ of the data are presented in Table 1, which shows that mean meditation level of the participants in the garden is the highest and the supermarket is the lowest. (Fig 9) shows a box plot comparing the mediation levels in the café, supermarket and the garden. It is clear from the figure that the mediation levels are higher in the café and the garden from that in the supermarket.

**Table 1:** Meditation level means and standard deviations.

Place (P)	Mean (m)	Std. Deviation (σ)	N
Café	56.9777	5.86631	10
Supermarket	48.7153	6.60941	10
Garden	60.5617	5.93503	10

To prove this assumption, we carried out One-Way ANOVA test [42], on meditation data for each place (café Pc, supermarket Ps, and garden Pg). ANOVA test is a statistical model used to determine whether there are any statistically significant differences between the means of three or more independent groups.

The test employs Wilks’ Lambda λ distribution which is a probability distribution used in multivariate hypothesis testing [43][44]. The distribution is defined by the parameter λ, which is given by:

$$\lambda =\frac{\det\left(S_{E}\right)}{\det\left(S_{H}+S_{E}\right)}=\prod_{k=1}^{m}\frac{1}{\left(1+\lambda_{k}\right)}$$ (1)

where *SE* represents the error matrix, SH is the hypothesis matrix and m is the number of meditation levels collected from each place. Our Null hypothesis suggests that all places (Pi) are equal in terms of meditation levels:

$$H_{0}:\;P_{c}=P_{s}=P_{g}$$

Conducting the ANOVA test has shown that, we reject the null hypothesis. The test results suggested that a place has a significant impact on meditation levels of the participants. Wilks’ Lambda λ = 0.199, F-value (2, 8) = 16.107, p-value <0.001. The results prove that we have an overall significant difference in means, but we do not know where those differences occurred. Table 2 is used to discover which specific means differed. Post-hoc test was carried out using Bonferroni correction which is a multiple-comparison correction used when statistical tests are being performed at the same time [45]. The test calculates the mean differences in order to find significant variances among places. Negative values in these differences indicate lower meditation mean in the first place. The pairwise comparison test revealed that there was no significant difference in the meditation levels in the café and supermarket since p = 0.053>0.05.

Furthermore, the difference between the café and garden meditation levels were not statistically significant (p = 0.855). However, the test indicated that there was a significant difference in the meditation levels between the supermarket and garden; (p <0.001). The boxplot in Fig 9 shows systematic differences in the meditation levels in different places.

**Table 2:** Pairwise comparison test.

(I) Place	(J) Place	Mean Difference (I-J)	Std. Error	p-value
Café	Supermarket	8.262	2.847	.053
	Garden	-3.584	3.153	.855
Supermarket	Café	-8.262	2.847	.053
	Garden	-11.846	2.058	**.001**
Garden	Café	3.584	3.153	.855
	Supermarket	11.846	2.058	**.001**

Bold values indicate p-values less than the significance level (0.05)

### 5.3 Mental states classification

Two classification algorithms were employed in this work. Naïve Bayes and J48 Decision Trees [46][47], which are commonly used in the literature to classify mental states associated with different tasks.

The same data collected in the three places were used for labelling. The labels were collected during a post-experiment questionnaire that asked the participants to tag their mental states in each place. The participants had to specify one of three mental states at each place, these are: Relaxed, Stressed or Neutral. In addition to the places data, another dataset was collected using Neurosky EEG headset for training purposes in the classification model. In this dataset, the subjects were instructed to wear the EEG headsets to record brain signals while experiencing two different mental states.

Data collected by the Neurosky EGG headset includes raw EEG, frequency bands, attention and meditation levels, and eye blinks. The collected data were pre-processed and cleaned prior to any analysis and classification. The beginning and the end of each recording were cropped which are highly prone to movement artifacts. Data were also partitioned into “10 seconds” segments. Data Segmentation is an essential step to improve classification accuracy and develop efficient systems. The “10 seconds” duration was calculated based on several trials. The following statistical features are calculated for each segment: mean, standard deviation, quartiles, quartile deviation, and signal derivative computed over each time period.

The performance of the models was evaluated using two standard metrics; both classification accuracy and kappa statistic were chosen to evaluate the performance of the classifiers.

“Kappa statistic” is a correlation coefficient to measure the agreement in categorical data [48] which is calculated using the following equation:

$$K=\frac{P(A)-P(E)}{1-P(E)}$$ (2)

where P(A) is the percentage agreement (i.e. the average True-Positive rate) and P(E) is the chance agreement. Its value is zero for the lack of any relation and approaches to one for very strong statistical relation between the class label and attributes of instances.

Table 3 shows the classification accuracies and Kappa statistic results using Ten-fold cross-validation. Bayes shows slightly better performance than decision trees, with 90% classification accuracy.

**Table 3:** Classification accuracy using 10-folds cross validation evaluation technique.

10-Folds-Cross validation	Naïve Bayes	J48 Decision Trees
Mean Classification accuracy ($\bar{x}$)	90%	86.6667%
Mean Classification error ($\bar{x}$)	10%	13.3333%
Kappa statistic	0.80	0.7333

In an attempt to enhance the performance results of classifiers, a set of the most effective features are required to be selected using feature selection methods.

A huge number of algorithms for feature subset selection have been proposed in the literature [49], [50], including sequential floating forward selection (SFFS), sequential forward selection (SFS), sequential backward selection (SBS). Feature selection methods use a subset evaluator that creates all possible subsets from the feature vector. Most feature selection methods use a criterion based on a specific classifier and are therefore useful if the classifier to be used is already known. Since the performance of most of these selection algorithms is strongly dependent on the given data set (and often relies on trial-and-errors).

We adopted Best First Search (BFS) algorithm which performed slightly better than the other algorithms. The technique was applied on our features based on both Naïve Bayes and J48 algorithms. Feature selection on Naïve Bayes showed that High Alpha and Meditation Level are the most effective features, while with J48 Decision Trees algorithm, the evaluator have shown that High Alpha, High Beta, Low Gamma and Meditation Level features are the most effective features and that gives the best classification results possible. Table 4 shows the classification results after selecting the most effective features for both algorithms. By comparing these results with the classification results before applying the feature selection technique, we notice that Naïve Bayes performance has improved slightly better in terms of classification accuracy than J48 Decision Trees.

**Table 4:** Classification accuracies with attribute selection.

10 folds-Cross valid	Naïve Bayes	J48 Decision Trees
**Mean Classification accuracy**($\bar{x}$)	90.8333%	86.6667%
**Mean Classification error** ($\bar{x}$)	9.1667%	13.3333%
**Kappa statistic**	0.8167	0.7333

After applying different machine learning algorithms on the labelled mental states dataset, another dataset containing the mental states tagged with different places was tested.

Na**ï**ve Bayes and J48 Decision Trees were also tested on a supplied test set since the results shown are the best results among the other learning algorithms. The external data set contains 10 seconds segments of the data collected from different places. The café data instances were labelled as ‘‘Relaxed”, the supermarket as ‘‘Stressed”, and the garden as ‘‘Relaxed” as tagged by the participants. The Bayes algorithm achieved $\bar{x}$ = 70.8% mean classification accuracy and Kappa statistic value of KDT = 0.4067 which indicates the existence of moderate statistical dependence. Different trials were made to improve the classification accuracies obtained from the external test set (subjects data set), but the enhancements noted were very slight. The results shown here are the best results possible even after applying attribute selection.

The second model utilizes J48 Decision Tree algorithm. The external dataset also contains three places with subject-tagged mental states. The J48 algorithm achieved $\bar{x}$ = 61.3% mean classification accuracy and Kappa statistic value of KDT = 0.2648, as shown in Table 5. The results indicate that only 61.3% of the mental states detected at the three places were correctly predicted. The Kappa statistic value indicates a low statistical dependence. From these results, we notice that the classification accuracies are subject-dependent, since some of test sets show high classification accuracy and others show poor accuracies.

**Table 5:** Classification accuracy using supplied test set evaluation technique.

Supplied Test set	Naïve Bayes	J48 Decision Trees
Mean Classification accuracy ($\bar{x}$)	70.8888%	61.3332%
Mean Classification error ($\bar{x}$)	29.111%	38.5110%
Kappa statistic	0.4067	0.2648

In order to classify places, the results of the classifiers described above are utilized to identify mental states at the three chosen places. As mentioned before, the classifiers were trained to recognize places based on the mental state detected by participants. Therefore, to classify places, classification errors produced by the Na**ï**ve Bayes classifier are utilised to evaluate the performance, when predictions were calculated. Mean Absolute Error (MAE) is used to assess prediction errors and to evaluate the variations in the errors in a set of predictions. In this work, MAE measure is used to calculate errors in mental states predictions.

MAE measures the average magnitude of the errors in a set of predictions. The Mean Absolute Error is given by:

$$MAE=\frac{1}{n}\sum_{i=1}^{n}\left|Pi-Ai\right|$$ (3)

where n is the number of data instances, *Pi* is the prediction probability and *Ai* is the actual value.

Table 6 shows the Mean Absolute Errors in predicting mental states at each place. It is evident that café is showing the highest mean error $\bar{x}$ = 0.3291in predicting the mental state in the café as ‘‘Relaxed”. Supermarket has MAE of only $\bar{x}$ = 0.23614 in predicting Supermarket as ‘‘Stressed”. In addition, garden prediction errors are low with MAE of $\bar{x}$ = 0.2947. These results show evidence of higher mental changes at the café than the other places, since some people may become intermittently stressed in the café environment. However, the error levels in mental states predictions are considered low which ensures that the actual mental states sensed at the places were correct. And hence, places can be classified according to the mental states of the individuals (i.e. ‘‘Relaxing” or ‘‘Stressful”).

**Table 6:** MAE in mental states predictions.

Place	Café	Supermarket	Garden
Actual State	‘‘Relaxed”	‘‘Stressed”	‘‘Relaxed”
Mean MAE $\bar{x}$	0.3291	0.23614	0.2947

**Fig 10:**
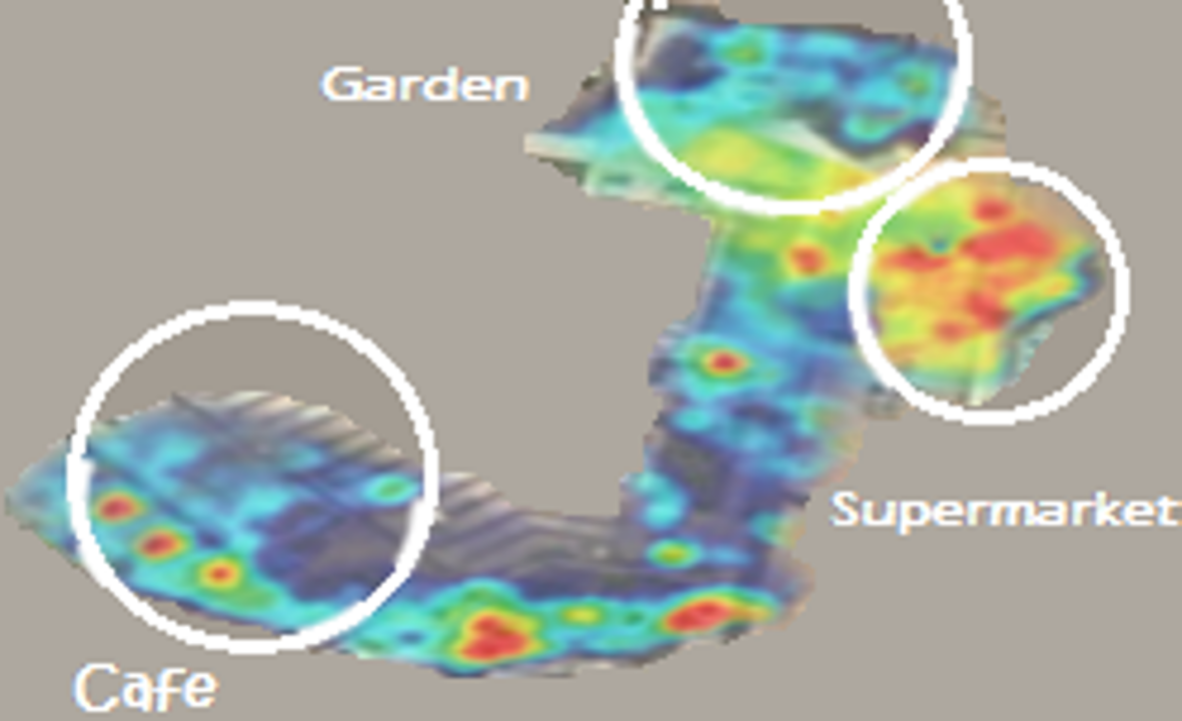
Heatmap of stress levels in three places (cafe, supermarket and garden).

Fig 10 shows a heat map of the three places and the subjects’ stress levels. It is clear that the garden has demonstrated lower stress levels, while the supermarket showed higher levels of stress. The café is exhibiting moderate stress and meditation levels since some of the subjects were stressed and were in a meditation mental state.

## 6 Discussion

To our knowledge this is one of the first research projects to utilise a mobile phone and EEG headset to classify urban places according to mental states. The experiments and results exhibited a noticeable change in mental states in relation to different places based on input from EEG signals. These differences allowed us to understand the impact of urban environments on mental states. By visually inspecting EEG data, meditation levels were found to be high in places such as café and garden, while low meditation levels in the supermarket environment. In addition, frequency bands associated with relaxation mental state such as alpha α band was noticed in these places. Beta β band is associated with mental activity and it was observed in supermarket where high mental activity is required.

The statistical analysis presented earlier in section 5.3 showed an agreement with the classification results. Performing post-hoc test on the places categories, suggested that the difference between café and supermarket, and between café and garden was not statistically significant in relation to meditation levels. However, the test has found the differences between meditation levels between supermarket and garden. Statistically significant. This is in line with environmental restorative theory which links natural and green areas to relaxation and tranquillity.

Furthermore, the classification results presented in Table 4 have shown a promising nature of our brain activity-based mental state recognition. Despite noisy labels and difficulties in recording users data in the ‘‘wild”, our mental states inference is still able to achieve %90 accuracy levels.

This work presents classification of mental states in outdoor environments based on EEG signals. Dataset containing training data of two mental states using ten-second segments was tested to evaluate different learning algorithms and then build a model to be used on our testing data.

The obtained results from the external test sets using the two algorithms have achieved lower accuracies than the mental states training set. The results obtained using the external test sets show high divergences among subjects. In both models, the classification accuracies vary depending on the subject. Thus, building systems that classify mental tasks and states is highly subject-dependent and require further analysis in the future. Based on the classification results, places were classified into ‘‘Relaxing” and ‘‘Stressful” environments and then visualized on a map to guide people to tranquil places that can relieve stress and mental exhaust.

## 7 Limitations

One of the challenge in monitoring brain activity in urban environment is to scale up the work and conduct large studies with many subjects and also to measure more environmental and physiological variables to understand the overall relationship between city places and body responses. Beside EEG headset, future studies could include, air pollution or noise sensors, heart rate, temperature sensors and UV and motion sensors. Many of these sensors are available on mobile phones and on many of the commercial smart watches.

This will create a large dataset with multiple exposures and health responses, which cannot be analysed using simple machine learning models.

**Fig 11:**
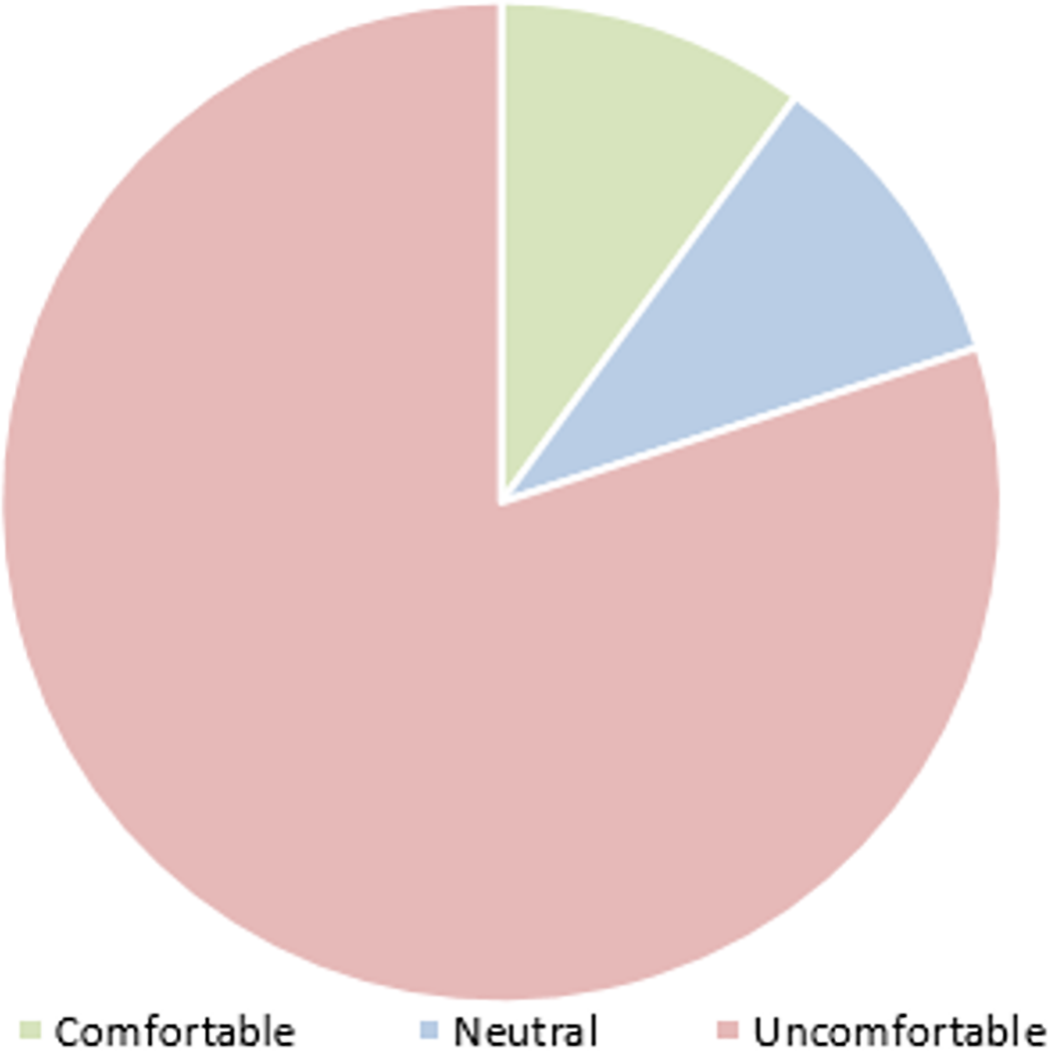
EEG headset comfort level.

The EEG headsets usually contain a number of electrodes which are metallic sensors usually placed on the head. Wearing the headset for long time could hurt or disturb people. Most of our participants have faced some level of discomfort while wearing the headset according to a post experiment questionnaire. (Fig 11) shows that 80% of the subjects experienced some degree of discomfort caused by the EEG device. Through our experiments we have noticed that, our participants have become increasingly apprehensive about what data are being collected about them, some users have expressed their concerns about the headset ability in reading their thoughts. However, these devices can only detect the mental activity as mentioned earlier.

In general users who wear brain sensing devices usually need extra assurance that their privacy is safeguarded. It would be problematic if their emotions and personal information are shared with others without their consent. With this in mind the *NeuroPlace* system is designed to collect users’ sensor data without associating it with users’ personal details.

## 8 Enabled applications

This work support previous effort in utilising wearable sensors for emption analysis in the wild [51–59], in particular, the analysis of EEG data in urban outdoor places opens a window for new applications including:

- **Attention restoration theory**: worries and stress about jobs, money and the hectic pace of the modern life need to be relieved. Different technologies can be used to monitor changes and improvements in restoring attention in individuals who suffer from attention fatigue. Our work presents one of the applications of using EEG technology to monitor attention restoration in favourite places.
- **Eco-therapy** (also known as nature therapy): contact with nature is energetic and therapeutic for both body and soul and can improve mood and ease anxiety, depression and stress. Mobile brain-sensing techniques such as NeuroPlace can provide health care providers with the means to track and test brain activities and hence recommend a relevant therapy.
- **Neuro-Marketing**: Advertisers have long used science to peer into consumers' brains. Today it is possible to monitor customers’ behaviour and mental activity using EEG to monitor mental activity around particular shopping zones. This enables marketers and advertisers to better understand the effectiveness of advertising, branding, product development, and packaging.
- **City navigation**: today, mobile devices are equipped with GPS which provides a basis for different location-aware applications. Many people are using maps available on their mobile phones to help them navigate urban areas. Sensing urban spaces (e.g. taking photos or using special sensors) improve users’ perception of the city. Geo-tagging urban areas with mental states can be utilized in navigation and tourism industry.
- **Neuro-feedback**: is direct training of brain function, by which the brain learns to function more efficiently. By observing the brain in action from moment to moment, then showing that information back to the person. And then rewarding the brain for changing its own activity to more appropriate patterns. NeuroPlace helps in discovering the impact of place on brain activities and hence recommending a suitable neuro-feedback in the form of games to heal and entertain individuals on the move.
- **Urban Planning and Smart City Analytics**: It is crucial for urban planners and decision-makers to listen to citizens' opinions regarding their local environments. This is an essential requirement in the design and creation of smart cities. Combining smart sensors and mobile phones that is capable of recording users' ratings, emotions, mental states as well as their locations are becoming very popular. Specifying which places are more stressful or relaxing is important in urban area planning. These are all enabling technologies for urban planning and decision making.
- **Mobile Crowdsourcing**: The proposed system can be used for data collection from the crowd, known as crowdsourcing. The mobile crowdsourcing approach is a data collection method used to collect data from different users in different places.

## 9 Conclusion and future work

Every human perceives outdoor spaces differently. Some places are seen to be hectic and stressful, others are perceived as tranquil and pleasant. This perception is subjective and emotions are mapped in the brain as direct reflection of the real physical map.

In this work, we presented NeuroPlace as an effective categorizing system to classify outdoor places according to the current mental states with a focus on relaxation, that is, where brain-waves’ readings become more meditative to assist people in restoring attention and relieving stress. We explored the properties and temporal structures of the EEG signals associated with place stimuli to distinguish places types. NeuroPlace opens up new opportunities and challenges in pervasive computing and mobile sensing research domains. This work demonstrated the advantages of using the EEG technology in exploring people mental perception of tranquil and relaxing environments. The results and findings of this research offer a wide range of future research opportunities and possibilities. The work presented in this paper represents a starting point for a wide range of research exploring how wearable sensors can tune into the minds' activity, which helps to understand the surrounding environment. However, the low reliability and subject' variability in the data prevent a rapid deployment of this technology in real applications. More research efforts are needed for improving the Brain-Computer Interfaces (BCI) in order to offer real life applications that contribute to the people’s quality of life. These developments should ensure that they have a lasting impact on the society. In this paper, we identified a list of potential applications of these technologies such as Eco-therapy, neuro-marketing, City navigation and many others.

Future research work will include using more robust signal analysis for feature extraction from EEG signals. In addition, using more advanced machine learning techniques such as ensemble methods to improve the efficiency of the system. Moreover, the EEG signals can be integrated with other wearable sensors for enabling the previously presented applications in real world settings. There is also an interesting area of spatial visualisation of the EEG data, which reveals which parts of the brain is activated at some point.
